# Centrosome-Kinase Fusions Promote Oncogenic Signaling and Disrupt Centrosome Function in Myeloproliferative Neoplasms

**DOI:** 10.1371/journal.pone.0092641

**Published:** 2014-03-21

**Authors:** Joanna Y. Lee, Wan-Jen Hong, Ravindra Majeti, Tim Stearns

**Affiliations:** 1 Department of Biology, Stanford University, Stanford, California, United States of America; 2 Stanford Institute for Stem Cell Biology and Regenerative Medicine and Cancer Institute, Stanford University School of Medicine, Stanford, California, United States of America; 3 Department of Medicine, Division of Hematology, Stanford University School of Medicine, Stanford, California, United States of America; 4 Department of Genetics, Stanford School of Medicine, Stanford, California, United States of America; Institut de Génétique et Développement de Rennes, France

## Abstract

Chromosomal translocations observed in myeloproliferative neoplasms (MPNs) frequently fuse genes that encode centrosome proteins and tyrosine kinases. This causes constitutive activation of the kinase resulting in aberrant, proliferative signaling. The function of centrosome proteins in these fusions is not well understood. Among others, kinase centrosome localization and constitutive kinase dimerization are possible consequences of centrosome protein-kinase fusions. To test the relative contributions of localization and dimerization on kinase signaling, we targeted inducibly dimerizable FGFR1 to the centrosome and other subcellular locations and generated a mutant of the FOP-FGFR1 MPN fusion defective in centrosome localization. Expression in mammalian cells followed by western blot analysis revealed a significant decrease in kinase signaling upon loss of FOP-FGFR1 centrosome localization. Kinase dimerization alone resulted in phosphorylation of the FGFR1 signaling target PLCγ, however levels comparable to FOP-FGFR1 required subcellular targeting in addition to kinase dimerization. Expression of MPN fusion proteins also resulted in centrosome disruption in epithelial cells and transformed patient cells. Primary human MPN cells showed masses of modified tubulin that colocalized with centrin, Smoothened (Smo), IFT88, and Arl13b. This is distinct from acute myeloid leukemia (AML) cells, which are not associated with centrosome-kinase fusions and had normal centrosomes. Our results suggest that effective proliferative MPN signaling requires both subcellular localization and dimerization of MPN kinases, both of which may be provided by centrosome protein fusion partners. Furthermore, centrosome disruption may contribute to the MPN transformation phenotype.

## Introduction

Myeloproliferative neoplasms are a class of chronic leukemias and malignant bone marrow disorders characterized by abnormal proliferation of one or more of the myeloid lineages. One of the molecular mechanisms underlying the transformation of a normal blood cell to a malignant cell involves chromosomal translocation events which join segments of two otherwise separated genes, creating at least one new fusion gene whose function is associated with the transformed phenotype. The resulting leukemia-associated fusion proteins provide growth and survival advantages by interfering with regulation of differentiation, apoptosis, and proliferation [1]. The leukemia-associated translocations are classified by the type of regulatory protein making up one of the pairs in the fusion: fusions with transcriptional regulator genes are associated with acute myeloid leukemia (AML), whereas fusions with tyrosine kinase genes are associated with myeloproliferative neoplasm (MPN), formerly known as myeloproliferative disease (MPD) [2], [3].

The proteins identified as partners in the fusions with tyrosine kinases are varied in function, including proteins involved in intracellular trafficking, nuclear functions, and regulatory processes [4], [5]. However, one common theme is that many of the MPN fusion partners are proteins that localize to the centrosome [6]. The centrosome is the main microtubule-organizing center of animal cells. Each centrosome consists of two centrioles and associated pericentriolar material. The centrosome is involved in cell cycle progression, possibly by serving as a scaffold for signaling proteins [7], [8]. Additionally, the centrosome templates the growth of a primary cilium, which is found in many cell types in mammals and is required for several important signaling pathways. Mutations in ciliary signaling pathways such as Hedgehog (Hh) and PDGFRα are commonly found in cancers [9], [10]. Although blood cells have not been reported to form cilia, chronic myelogenous leukemia (CML), a form of MPN, has been shown to require Hedgehog signaling for survival of the leukemic stem cell population [11], [12]. Therefore, these cells must either possess some form of primary cilium or perform Hh signaling in the absence of a cilium; a process shown to require a cilium in other mammalian cell types [13].

What functions might a centrosome protein impart upon the leukemia-associated fusion protein? In all identified cases an N-terminal segment of the centrosome protein is fused to a C-terminal segment of a receptor tyrosine kinase (RTK) [6], [14]. RTKs typically contain an N-terminal extracellular regulatory domain, a transmembrane domain, and a C-terminal intracellular kinase catalytic domain. The leukemia-associated fusions retain the kinase domain but lack extracellular and transmembrane domains [4], [15], [16]. Upon ligand binding, receptors dimerize, resulting in kinase activation. Many of the partner proteins, including centrosomal partners, contain protein-protein interaction domains, which are thought to promote kinase dimerization and activation in the absence of regulatory domains [4], [5], [17]. Indeed, the presence of oligomerization domains in virtually every MPN fusion partner has been considered as evidence that dimerization is the only crucial role of the partner protein. However, centrosome proteins make up only 3.6% of total coiled-coil proteins (Marcoil prediction [18]) while they appear in almost half of MPN fusions. MPNs caused by protein fusions with FGFR1 have clinical presentations of disease that vary depending on the fusion partner [14], suggesting that partners play a role in generation of phenotype that is independent of presence of dimerization domains. One such role may be disruption of normal centrosome function. Centrosomes aberrations are frequently observed in cancers and abnormal γ–tubulin staining has been reported in CML patient cells [19].

Given the disproportionate number of partner proteins that share a common subcellular localization at the centrosome, the importance of this localization has previously been tested. Targeting of the PDGFRα and PDGFRβ catalytic domains to the centrosome using the PACT domain [20] did not enhance oncogenicity as assayed by IL-3 independent growth of BaF3 cells, a mouse bone marrow-derived cell line [21]. However, it is possible that dimerization is required in concert with localization.

In one type of MPN, the FGFR1 tyrosine kinase is fused with the centrosome protein FOP [15]. When the FOP-FGFR1 fusion protein is expressed in BaF3 cells, it localizes to the centrosome where it recruits and phosphorylates its signaling substrates [22], [23], [24]. Retroviral transduction of FOP-FGFR1 in primary blood cells reproduces MPN in mice [23]. In this study, we test the functional significance of centrosome fusion partners in MPN. We assay the contribution of centrosome partner proteins in transformative MPN fusion signaling. Additionally, we assay centrosome disruption in MPN fusion expressing patient samples and RPE-1 cells, both of which show centrosome defects.

## Results

### Separation of function mutations in FOP-FGFR1

**Figure 1:**
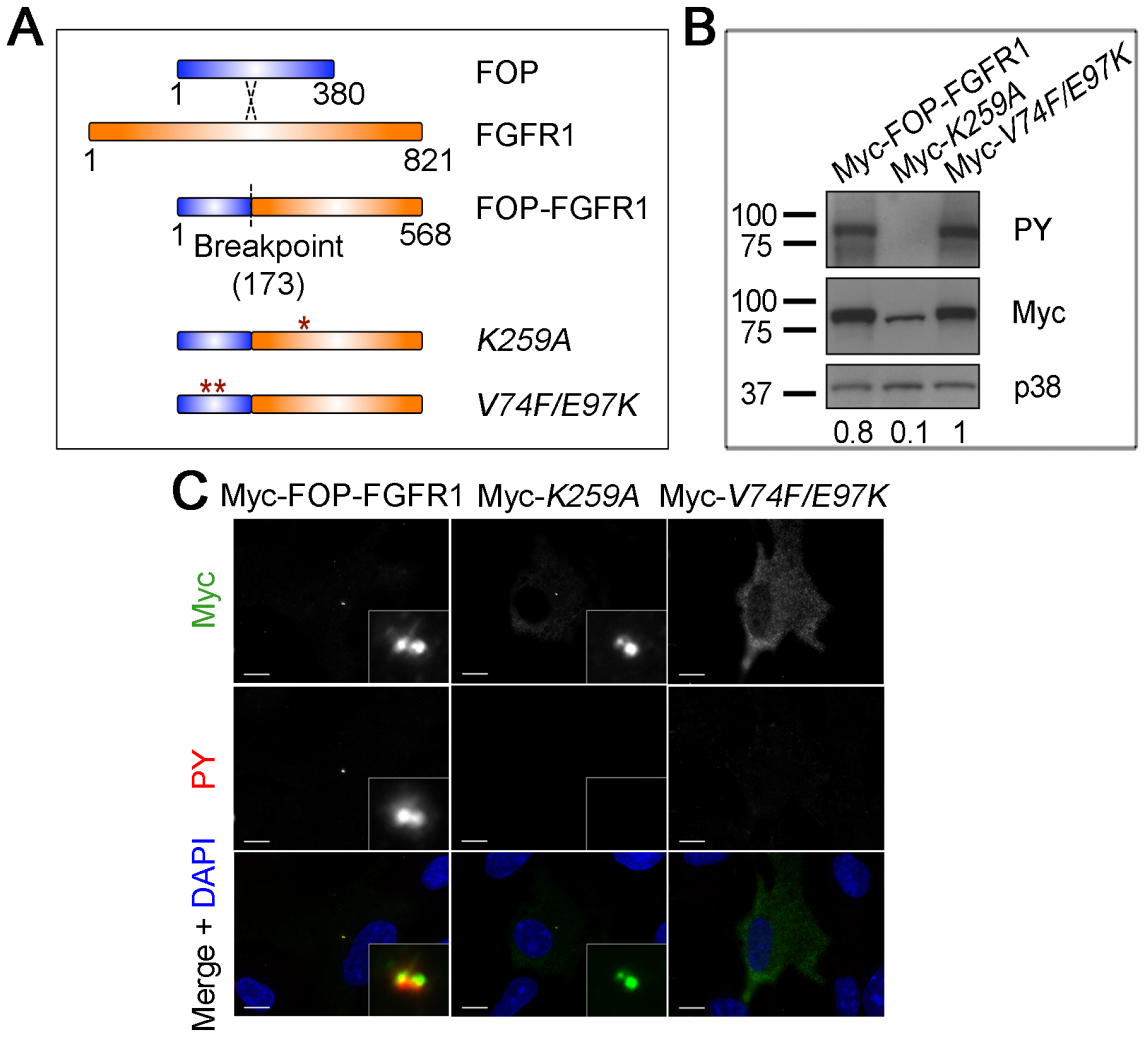
FOP-FGFR1*^V74F/E97K^* mutant lacks centrosome localization but not kinase activity. (**A**) Schematic of fusion between FOP and FGFR1 resulting in the FOP-FGFR1 oncogenic fusion and subsequent point mutations to produce KD, FOP-FGFR1*^K259A^*, and centrosome localization mutant, FOP-FGFR1*^V74F/E97K^*. (**B**) WB analysis of lysates from RPE-1 cells transfected with WT Myc-FOP-FGFR1, Myc-FOP-FGFR1*^K259A^*, or Myc-FOP-FGFR1*^V74F/E97K^*, harvested, and probed with antibodies against phosphotyrosine (PY), Myc, and p38 as a loading control. (**C**) RPE-1 cells transfected with Myc-FOP-FGFR1, Myc-FOP-FGFR1*^K259A^*, or Myc-FOP-FGFR1*^V74F/E97K^*, fixed, and stained with antibodies against Myc (green) and PY (red). DNA is stained using DAPI (blue). Scale bars: 10 μm; insets: 10× magnification.

To test the importance of centrosome localization in MPNs, we used the centrosome-localizing MPN fusion FOP-FGFR1 as a system. FOP-FGFR1 localizes to centrosomes, where it leads to increased phosphotyrosine (PY) labeling [22]. To test the importance of centrosome localization on MPN fusion function, we made *V74F/E97K* mutations in the FOP portion of FOP-FGFR1 that were previously shown to disrupt centrosome localization of FOP (Fig. 1A) [25]. A kinase-dead (KD) version of FOP-FGFR1, FOP-FGFR1*^K259A^*, has been previously described and was generated as a control (Fig. 1A) [22]. All constructs were expressed in RPE-1 cells, an hTERT-immortalized retinal pigment epithelial cell line. As previously reported, Myc-FOP-FGFR1 and Myc-FOP-FGFR1*^K259A^* both showed centrosome localization, co-localizing with glutamylated tubulin at centrioles (Fig. S1). Although we focus on FOP-FGFR1 at the centrosome proper, we note that a subset of cells also showed FOP-FGFR1 localization and PY labeling at centriolar satellites, small cytoplasmic particles associated with the centrosome, as we previously reported [26]. In contrast, Myc-FOP-FGFR1*^V74F/E97K^* localized to the cytoplasm with no detectable concentration at the centrosome (Fig. S1), demonstrating that the *V74F/E97K* mutations successfully disrupt centrosome localization of the FOP-FGFR1 fusion.

We then assayed the kinase activity of Myc-FOP-FGFR1*^V74F/E97K^*. The FOP*^V74F/E97K^* mutant protein was previously described as defective in dimerization as well as localization [25], [27]. As kinase fusion partners are thought to aid kinase activation by facilitating dimerization, FOP-FGFR1*^V74F/E97K^* could cause loss of fusion activity by disrupting dimerization. Co-immunoprecipitation (co-IP) experiments showed that the *V74F/E97K* mutations disrupt FOP-FGFR1 dimerization *in vitro* as reported for FOP*^V74F/E97K^*, however FOP-FGFR1*^V74F/E97K^* was able to co-IP differentially tagged FOP-FGFR1*^V74F/E97K^ in vivo* (Fig. S2), suggesting that it is capable of interaction in this context. The kinase activity of FOP-FGFR1*^V74F/E97K^* was tested directly by assaying for PY signal by western blot (WB) and immunofluorescence (IF) following expression and serum starvation in RPE-1 cells (Fig. 1B & 1C). Antibodies against PY recognize the autophosphorylated FGFR1 fusion proteins in addition to their tyrosine-phosphorylated substrates. Although RPE-1 cells normally express RTKs, PY by endogenous RTKs is low in the absence of growth-factor-containing serum. For this reason all PY assays are carried out under low serum conditions (0.5% serum) to reduce background from endogenous RTK signaling. WB analysis showed that Myc-FOP-FGFR1*^V74F/E97K^* has an amount of PY similar to that of WT Myc-FOP-FGFR1, which is absent for Myc-FOP-FGFR1*^K259A^* (Fig. 1B). In cells expressing FOP-FGFR1*^V74F/E97K^* there was diffuse cytoplasmic PY staining that corresponded with the cytoplasmic staining of the Myc-tagged construct, which was absent from Myc-FOP-FGFR1*^K259A^* controls (Fig. 1C). These results indicate that FOP-FGFR1*^V74F/E97K^* localizes to the cytoplasm as an active kinase.

**Figure 2:**
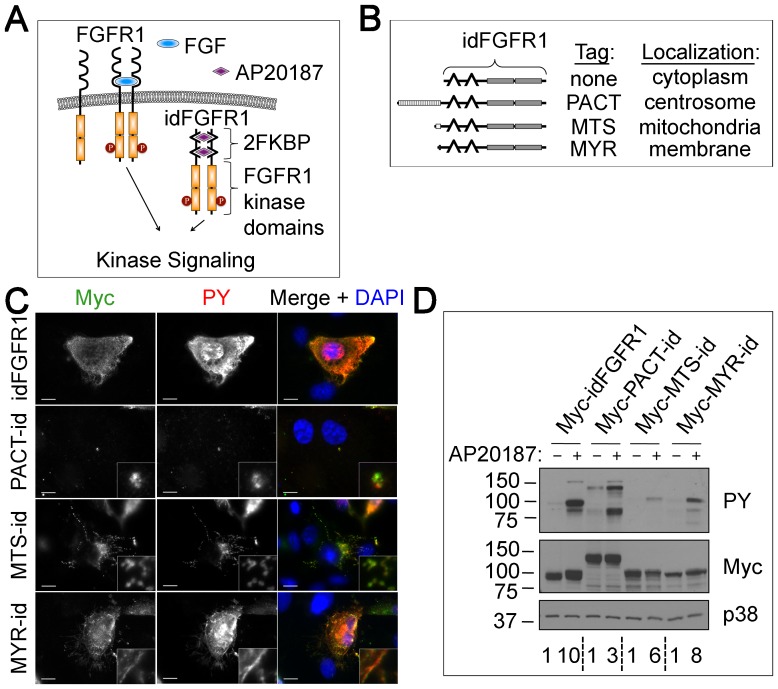
Subcellular targeting of inducibly dimerizable FGFR1 (idFGFR1). (**A**) Schematic of normal FGFR dimerization and idFGFR1 dimerization induced with dimerization ligand AP20187. (**B**) Table of idFGFR1 constructs targeted to subcellular domains by the addition of localization tags. (**C**) RPE-1 cells transfected with Myc-idFGFR1, Myc-PACT-idFGFR1, MTS-idFGFR1-Myc, or MYR-idFGFR1-Myc, treated with 10 nM AP20187 for 24 h, fixed, and stained with antibodies against Myc (green) and PY (red). DNA is stained using DAPI (blue). Scale bars: 10 μm; insets: 10× magnification. (**D**) WB analysis of lysates from RPE-1 cells transfected with Myc-idFGFR1, Myc-PACT-idFGFR1, MTS-idFGFR1-Myc, or MYR-idFGFR1-Myc, treated with 10 nM AP20187 or vehicle (100% ethanol) for 24 h in low serum medium, harvested, and probed with antibodies against PY, Myc, and p38 as a loading control. Quantification of PY increase relative to control, normalized by Myc, for each construct is indicated.

The previous constructs allowed us to test the importance of centrosome localization alone. To test the importance of centrosome localization in concert with kinase dimerization, we generated an inducibly dimerizable version of FGFR1 (idFGFR1), using the ARGENT regulated homodimerization system (ARIAD Pharmaceuticals, Inc.). idFGFR1 contains the truncated portion of FGFR1 retained in MPN FGFR1 fusions, with the addition of FKBP domains on the N-terminus (Fig. 2A). Intermolecular dimerization of FKBP domains is achieved by addition of dimerization ligand AP20187. Targeted localization of idFGFR1 was achieved through addition of localization tags.

We considered that whatever function provided to the FOP-FGFR1 fusion protein by centrosome localization might be similarly fulfilled by localization to cell structures other than the centrosome. To account for this possibility, constructs were made in which either FGFR1 or dimerizeable idFGFR1 would be targeted to the centrosome, mitochondrial membrane, and plasma membrane through addition of the PACT domain [20], a mitochondrial targeting sequence (MTS) [28], and a myristoylation sequence (MYR) [29], respectively (Fig. 2B). Expression of these constructs showed that each localized as expected from the localization module in the construct, and that the amount of PY was markedly increased at those locations in the presence of AP20187 (Fig. 2C). The dimerizable idFGFR1s were also tested for kinase activation by assaying the amount of kinase autophosphorylation, measured by the amount of PY signal at the position of the Myc-tagged construct on a WB (Fig. 2D). These results suggest that targeted constructs localize properly and that addition of dimerization ligand promotes construct kinase activation.

We considered that increased local concentration could promote spontaneous kinase dimerization even in the absence of intrinsic dimerization ability. We tested this by assaying kinase activation of targeted constructs in the absence of drug. In each case, a band corresponding to the idFGFR1-bearing construct showed an increase in PY labeling upon the addition of AP20187 (Fig. 2D), suggesting that construct targeting alone does not generate appreciable kinase dimerization. Importantly, Myc-PACT-idFGFR1 showed moderate amounts of PY even in the absence of AP20187. This is likely a result of the intrinsic dimerization ability of the PACT domain. In agreement, co-IP experiments using *in vitro* translated protein showed dimerization of PACT-cFGFR1, a construct containing PACT fused to the truncated portion of FGFR1 found in MPN without the addition of FKBP dimerization domains (Fig. S3). However, experiments using *in vitro* translated PACT-idFGFR1 showed that addition of AP20187 resulted in an increase in dimerization (Fig. S3) consistent with the increased kinase activity in *in vivo* experiments (Fig. 2D). This suggests that increasing local kinase concentration by targeting does not promote kinase dimerization, however addition of the PACT domain alone does result in some kinase activation due to the intrinsic dimerization ability of PACT.

### Efficient PLCγ phosphorylation requires FGFR1 dimerization and localization

**Figure 3:**
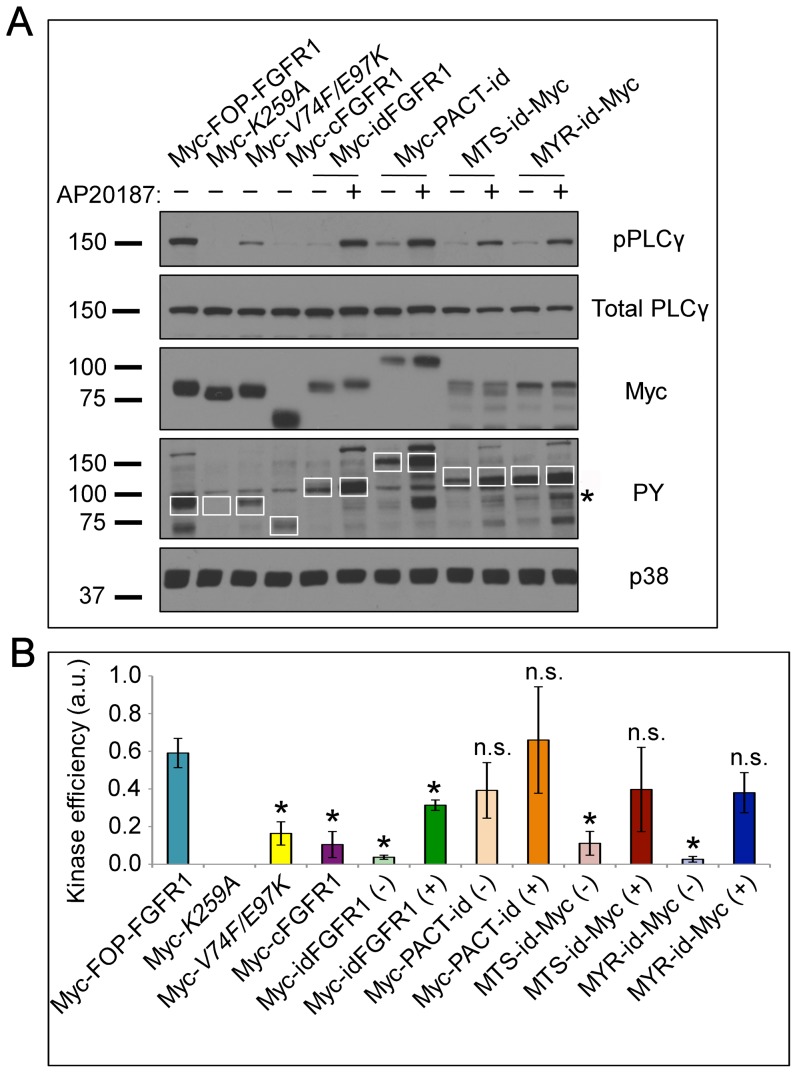
Dimerization and subcellular targeting are required in FGFR1 MPN signaling. (**A**) WB analysis of lysates from RPE-1 cells transfected with Myc-FOP-FGFR1, Myc-FOP-FGFR1*^K259A^*, Myc-FOP-FGFR1*^V74F/E97K^*, Myc-cFGFR1, Myc-idFGFR1, Myc-PACT-idFGFR1, MTS-idFGFR1-Myc, or MYR-idFGFR1-Myc, treated with 10 nM AP20187 or vehicle (100% ethanol) for 24 h in low serum medium, harvested, and probed with antibodies against phospho-PLCγ (pPLCγ), total PLCγ, Myc, PY, and p38 as a loading control. White boxes indicate quantified regions in PY blot, * marks a non-specific band present in all lanes. (**B**) Graph showing kinase efficiency of each construct with or without AP20187 addition. Kinase efficiency  =  WB signal intensity of (pPLCγ/PLCγ)/PY. Quantifications were obtained using a Typhoon imaging system and fluorescence-conjugated secondary antibodies. Bars represent mean of 3 independent trials ± SEM. *p<0.05, n.s. p>0.05.

In the experiments above we generated the tools to allow us to test the relative contributions of centrosome localization, localization more generally, and dimerization to fusion-FGFR1 signaling. As a readout for the function of each construct we chose to assess the phosphorylation of PLCγ, a signaling substrate required in FOP-FGFR1-induced transformation (Fig. 3) [24], [30]. A construct containing the truncated portion of FGFR1 (cFGFR1) found in MPN fusions, without localization or dimerization domains, was included as a control. Constructs were expressed in RPE-1 cells and exposed to AP20187 or vehicle for 24 h before harvesting for WB analysis (Fig. 3A). The relative signaling effectiveness of each construct was measured by the fluorescence intensities of phospho-PLCγ (pPLCγ), total PLCγ, and PY of the fusion protein (Fig. 3A, white boxes) for each sample; the ratio of (pPLCγ/total PLCγ)/PY was used to calculate kinase signaling efficiency (Fig. 3B). This shows the percent of total PLCγ phosphorylated per unit of active kinase, thus accounting for variation in the total available PLCγ and differences in dimerization strength as a result of subcellular targeting. Construct expression level was determined by the Myc epitope tag on each construct and p38 was used as a loading control.

To test the relative contributions of localization and dimerization to kinase signaling, we compared the kinase signaling efficiencies of the various FOP-FGFR1 and targeted idFGFR1 constructs. Combination of induced dimerization with subcellular targeting to any site tested was sufficient to generate a signaling efficiency statistically indistinguishable from WT (Fig. 3). Undimerized idFGFR1 consistently had a decrease in kinase efficiency, regardless of targeted localization, indicating the prime importance of dimerization. However, localization to a discrete subcellular site also had an effect; cytoplasmically localized, dimerized cFGFR1 (both FOP-FGFR1*^V74F/E97K^* and dimerized idFGFR1) showed a statistically significant decrease in kinase efficiency. Importantly, addition of AP20187 to cFGFR1 lacking a dimerization domain had no effect on signaling efficiency. These results suggest that dimerization alone, but not localization, is sufficient to produce limited FGFR1 kinase signaling efficiency. However recapitulation of WT FOP-FGFR1 kinase efficiency requires localization to a surface or structure in addition to dimerization. Our results also show that the benefits of localization are not limited to the centrosome, as dimerized constructs targeted to the mitochondrial membrane or plasma membrane had a similar effect.

### FOP-FGFR1 expression in RPE-1 cells causes a defect in centrosome function

**Figure 4:**
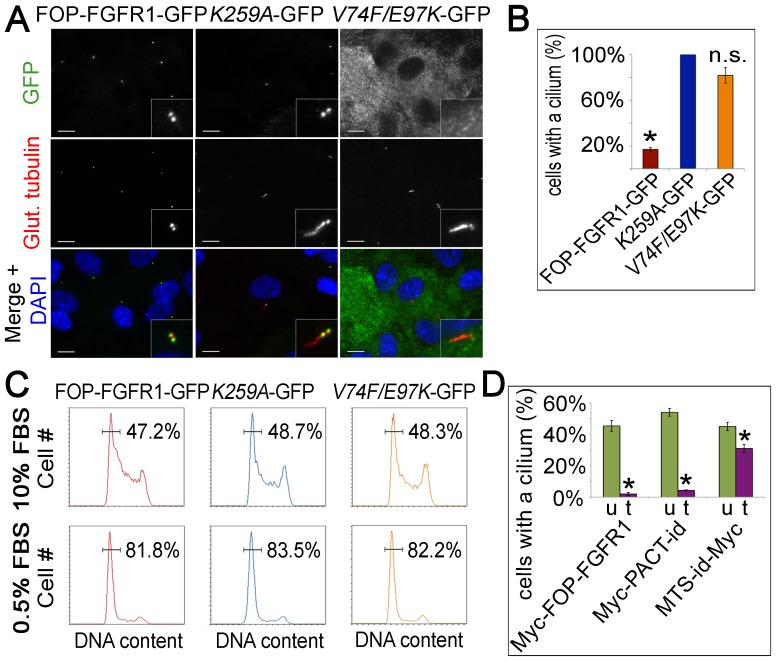
MPN fusion expression causes a decrease in ciliogenesis. (**A**) RPE-1 cells stably expressing FOP-FGFR1-GFP, Myc-FOP-FGFR1*^K259A^*-GFP, or Myc-FOP-FGFR1*^V74F/E97K^*-GFP, serum-starved for 48 h, fixed, and stained with antibodies against GFP (green) and glutamylated-tubulin (red). DNA is stained using DAPI (blue). Scale bars: 10 μm; insets: 5× magnification. (**B**) Graph showing percent of FOP-FGFR1-GFP and mutant expressing cells that form a primary cilium normalized to FOP-FGFR1*^K259A^*-GFP. Bars represent mean of three independent trials ± SEM. *p<0.05, n.s. p>0.05. (**C**) Percentage of GFP-positive cells with G_0_/G_1_ DNA content following 48 h incubation in complete or low serum medium. N = 10,000 cells each. (**D**) Graph showing percent of transfected (t) Myc-FOP-FGFR1, Myc-PACT-idFGFR1, and MTS-idFGFR1-Myc expressing cells that form a primary cilium compared to neighboring untransfected (u) cells following 48 h incubation in low serum medium containing dimerization ligand. Bars represent mean of technical triplicates ± SEM. *p<0.05.

In the assay above testing signaling efficiency of the fusion kinases, localization was shown to be important, but there was little difference between localization of active kinase to the centrosome and localization to other sites. Another possibility to explain the prevalence of centrosome protein fusion partners in MPN is that localization of the active FGFR1 kinase to the centrosome interferes with centrosome function in a manner beneficial to the cancer cell. We examined several aspects of centrosome function including microtubule nucleation, centrosome duplication and primary cilium formation. Although nucleation and duplication were unaffected, primary cilium formation was strongly decreased in FOP-FGFR1 expressing cells (Fig. 4A). RPE-1 cells stably expressing FOP-FGFR1-GFP, Myc-FOP-FGFR1*^K259A^*-GFP, or FOP-FGFR1*^V74F/E97K^*–GFP were serum-starved and assayed by IF for cilium formation. Expression of FOP-FGFR1-GFP resulted in an 83% decrease in the number of cells that formed a cilium when compared to KD FOP-FGFR1*^K259A^*-GFP. In contrast, cells expressing FOP-FGFR1*^V74F/E97K^*–GFP, which does not localize to the centrosome, showed only an 18% decrease in ciliogenesis (Fig. 4B).

As the ciliation assay was performed under low serum to enrich for cells in G_0_/G_1_, a decrease in ciliation may reflect defects in cell cycle arrest rather than ciliogenesis. To determine if FOP-FGFR1 expressing cells are responsive to low serum, DNA content was assayed by flow cytometry under normal and low serum conditions (Fig. 4C). Cells expressing FOP-FGFR1-GFP, FOP-FGFR1*^K259A^*-GFP, or FOP-FGFR1*^V74F/E97K^*–GFP arrested in G_0_/G_1_ equally well upon serum starvation, thus, the ciliogenesis defect is not due to a general cell cycle defect. RPE-1 cells transfected with Myc-PACT-idFGFR1 and incubated in low serum medium containing dimerization ligand also showed a substantial decrease in ciliogenesis (Fig. 4D & Fig. S4). Interestingly, kinase targeting to other subcellular locations had a smaller, but significant, effect on cilium formation (shown for MTS in Fig. 4D & Fig. S4). These results show that localization of active FGFR1 to the centrosome causes a strong defect in a key centrosome function, but kinase targeting to mitochondria also causes a centrosome defect, albeit to a lesser degree. As cytoplasmically localized kinase did not have the same effect, this suggests that indiscriminate subcellular targeting may play a role in MPN induced centrosome disruption, perhaps through kinase signaling.

### Centrosome defects in MPN patient cells

**Figure 5:**
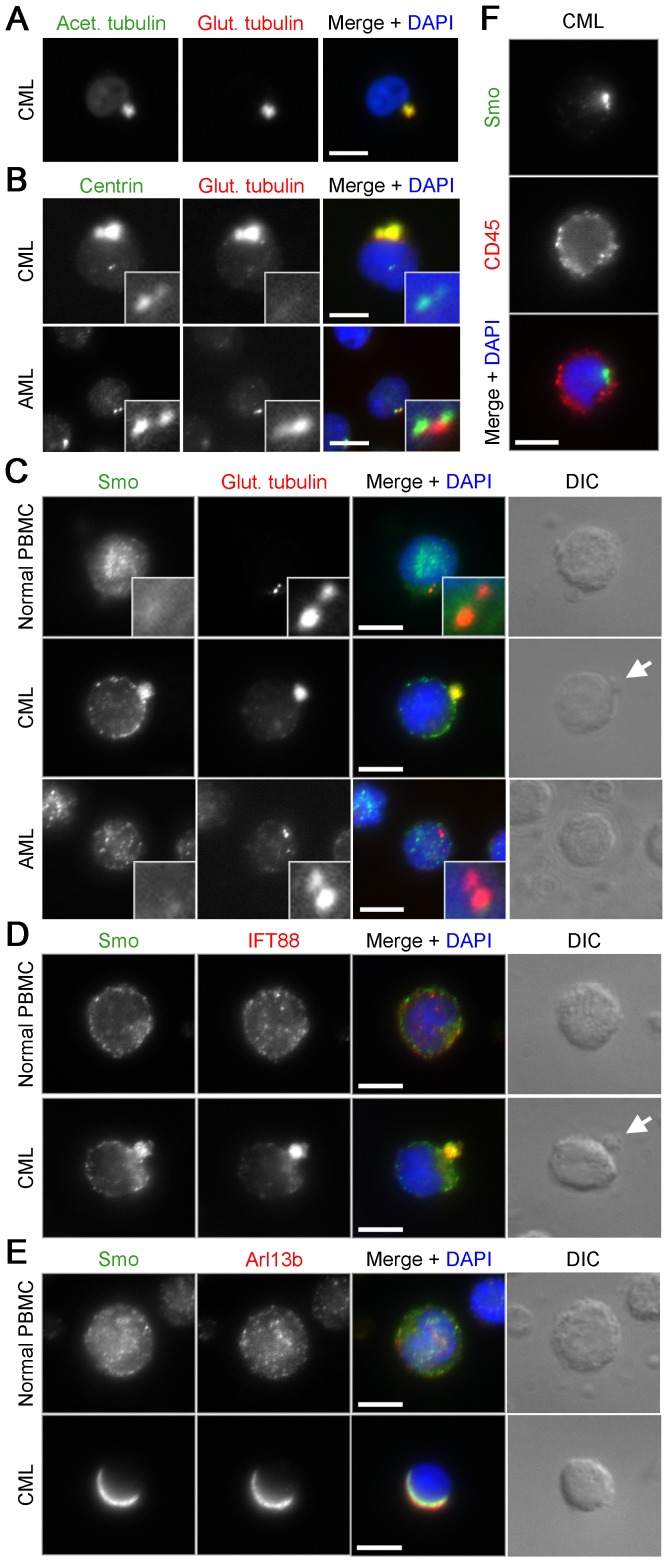
Localization of ciliary proteins in human CML cells. (**A**) Human CML cells stained with antibodies against acetylated (green) and glutamylated tubulin (red). (**B**) CML and AML cells stained with antibodies against centrin (green) and glutamylated tubulin (red). (**C**) Normal PBMC, CML, and AML cells stained with antibodies against Smoothened (Smo) (green) and glutamylated tubulin (red). (**D**) CML cells stained with antibodies against Smo (green) and either IFT88 (red). (**E**) CML cells stained with antibodies against Smo (green) and Arl13b (red). DNA is stained using DAPI (blue), scale bars: 5 μm, insets: 10× magnification, white arrows: cell protrusions. In each case >100 cells were imaged, and the phenotype represented in the images shown was seen in greater than 50% of cells.

The above results suggest that centrosome function can be compromised by activation of FGFR1, most strongly when targeted to the centrosome, but to lesser degree when targeted elsewhere. To test whether disruption of centrosome structure/function might be a common feature of MPN fusion expression, even in cases that do not involve known centrosome protein fusion partners, we directly assayed human MPN cells for centrosome defects. We obtained peripheral blood mononuclear cells (PBMC) from patients with chronic myelogenous leukemia (CML), the most common form of MPN, caused by the BCR-ABL translocation. We stained PBMC from three CML patients (Table S1) with antibodies against glutamylated and acetylated tubulin, stabilized forms of tubulin normally found in centrioles and the cilium. Cells from CML samples contained unusual masses of glutamylated or acetylated tubulin (Fig. 5A) to which a centriole protein, centrin, also localized (Fig. 5B). In addition, centrin localized to two puncta likely to be centrioles (Fig. 5B). Importantly, these masses of modified tubulin were not present in either of two acute myeloid leukemia (AML) patient samples (Fig. 5B & Table S1), a form of leukemia distinct from MPN that is not associated with centrosome gene translocations. Primary AML cells showed normal centriole staining, as did normal PBMC (Fig. 5B,C). Remarkably, BCR-ABL does not involve a centrosome protein fusion partner, although centrosome aberrations in CML have been described previously [19], suggesting that centrosome disruption is achieved through an indirect manner. These results suggest that MPN fusion expression disrupts centrosomes through the formation of aberrant centrosome protein-containing masses.

To further assess centrosome-related disruption, we assayed the localization of Smoothened (Smo) in CML cells. Interestingly, CML cancer stem cells have been shown to require the cilium-localized Hh signaling component Smo for proliferation [11], [12], [31]. CML cells had large Smo-positive structures that colocalized with glutamylated tubulin, whereas PBMC and AML cells had only diffuse, cytoplasmic Smo staining (Fig. 5C). Two other proteins associated with primary cilium function colocalized with Smo and glutamylated tubulin foci in CML cells (Fig. 5D & Fig. S5): IFT88, a component of intraflagellar transport normally found on the ciliary axoneme [32], and Arl13b, a small GTPase found in the ciliary membrane [33]. Interestingly, these masses in CML cells were associated with protrusions in most cases (Fig. 5C,D & Fig. S5, arrows). In other cells, Smo localized in a polarized manner on the CML cell membrane (Fig. 5E & Fig. S5). Polarization of signaling-associated proteins to a domain of the plasma membrane was infrequently seen in cells containing protrusions, however polarization could be observed in cells with smaller protrusions (Fig. S5). Importantly, CD45, a cell surface maker on hematopoietic cells unassociated with centrosomes or cilia neither colocalized with the tubulin/Smo masses, nor had polarized localization to the plasma membrane (Fig. 5F). These results suggest that expression of BCR-ABL, a fusion protein that colocalizes with actin at the cell periphery [34], causes proteins associated with the centrosome and cilium to form large polarized structures. Although primary cilium-associated proteins are required for Hh signaling in mammals [13], blood cells have not been observed to form cilia. Recently, the immunological synapse of cytotoxic T cells has been proposed to represent a modified cilium [35]. Our results may provide further insight into cilium-associated signaling in these unciliated blood cells.

## Discussion

We have shown that, 1) kinase targeting and dimerization are required for MPN kinase signaling and both can be provided by a centrosome protein fusion partner, 2) centrosome disruption is dependent on kinase targeting to any subcellular location, and 3) MPN fusion expression disrupts centrosome function. These results suggest that centrosome disruption may be a more common feature of MPNs than previously appreciated, which may contribute to the myeloproliferative phenotype. The result that centrosome disruption can be caused by non-centrosome fusions is in agreement with the existence of naturally occurring non-centrosome MPN fusion partners, many of which have been shown to localize to other subcellular domains and possess oligomerization domains [4], [6]. However, our results suggest that centrosome fusion partners may generate a stronger centrosome phenotype, which may be advantageous in MPN pathogenesis.

Our finding that targeting to any subcellular location is required for kinase signaling was unexpected. Two possible explanations for the subcellular targeting in signaling are 1) decreased kinase mobility, and 2) increased substrate availability (Fig. S6). Mathematical modeling suggests that restriction of a kinase to a small subdomain combined with high motility substrates produces the greatest kinase-substrate signaling and cell sensitivity [36]. This is consistent with a previous study of primary cilium signaling in which increasing the area of the localization domain resulted in a decrease in signaling [37]. Alternatively, increased substrate phosphorylation by a localized kinase might be due to increased substrate availability in the targeted locations. It should be noted, however, that PLCγ, the FGFR1 substrate assayed here, is not reported to differentially localize to centrosomes or mitochondria, but does translocate to the plasma membrane in response to EGF stimulation [38].

We found that the centrosome-localizing PACT domain fused to FGFR1 is sufficient to mimic the effects of FOP-FGFR1. We note that this contradicts previous findings that addition of PACT to PDGFRα or PDGFRβ, kinases found in MPN centrosome-kinase fusions, is not sufficient to cause proliferation of BaF3 cells [21]. This discrepancy may be due to differences in experimental design. In the previous study PACT was fused to the C-terminus of the kinases as opposed to the N-terminus, contrary to our study. Given that dimerization domains provided by fusion partners are at the N-terminus of the kinase in all known MPN fusions [6], this may indicate that structural organization is important for proper kinase activation. Additionally, the dimerization strength of PACT may be sufficient to achieve a relevant increase in signaling with FGFR1, but not PDGFRα/β.

We report here that FOP-FGFR1 expression disrupts centrosome function as assayed by ciliogenesis in epithelial cells. This is most likely due to localization of the active FGFR1 kinase to the centrosome, rather than to dominant interference with FOP function because PACT-idFGFR1 had a similar effect, whereas MTS-idFGFR1 had a weaker effect. In our assays with FOP-FGFR1 we used ciliogenesis as a proxy for centrosome structure and function, but we note that mature cells of the blood lineage have not been observed to form a primary cilium. Thus, the ciliogenesis defect observed is not necessarily related to the disease phenotype, but could be a manifestation of centrosome disruption that is easily observed in epithelial cells; the same molecular mechanism might result in other manifestations of centrosome disruption in blood cells. Given that there is evidence for centrosome disruption caused by at least two quite different MPN-associated fusion proteins in two different cell types, we propose that such defects might be a more common feature of MPNs than previously appreciated. MPN-related centrosome disruption appears to require targeted fusion localization, though not specifically to the centrosome.

Remarkably, disruption of centrosome structure was observed in human CML cells expressing the BCR-ABL fusion protein, in the form of large centrosome and cilium protein-containing structures not seen in control or AML cells. Interestingly, CML cancer stem cells have been shown to require Hh signaling [11], [12]. Primary cilium-associated proteins are required for Hh signaling in mammals [13]; although blood cells have not been observed to form cilia, we report evidence for organized localization of ciliary proteins in CML cells. Given that there is evidence for centrosome disruption caused by at least two quite different MPN-associated fusion proteins, we propose that such defects might be a common feature of MPNs, unique to this type of leukemia. This observation might be relevant to future therapies for CML and other MPNs.

## Materials and Methods

### Ethics Statement

Human samples were obtained from patients at Stanford University Medical Center according to the Institutional Review Board (IRB) approved protocols (Stanford IRB no. 6453). Participants provided written consent to participate in this study. The IRB approved this consent procedure.

### Human Samples

Human CML and AML PBMC were cryopreserved in liquid nitrogen in 90% FBS and 10% DMSO. Freshly thawed cells were fixed in 4% PFA, spun onto polyethyleneimine coated coverslips, and blocked for 1 h with guinea pig IgG in 3% BSA (Sigma) in PBS +0.1% Triton (PBS-BT) at 1∶500 for immunostaining.

### Plasmids

cDNAs for human FOP (GenBank: BC011902.2) and FGFR1 (GenBank: BC015035.1) were obtained from Open Biosystems. The FOP-FGFR1 fusion was generated using precise gene fusion by PCR [39], joining the first 519 nucleotides of FOP with the last 1,185 nucleotides of FGFR1 (cFGFR1). PCR products were cloned into pDONR221 using the Invitrogen Gateway system. The FOP-FGFR1*^K259A^* mutant was generated using site-directed mutagenesis and FOP-FGFR1*^V74F/E97K^* generated using site-directed mutagenesis and overlapping PCR following the V74F/E97K mutations reported to disrupt centrosome localization in FOP [25]. Synthetic MPN constructs were cloned using the PACT domain from pericentrin [20], FKBP domains (2× FKBP_36V_) PCR-amplified from pC4-Fv1E (ARGENT Regulated Homodimerization Kit Version 2.0; ARIAD Pharmaceuticals, Inc.), the MTS of human TOMM20 (NM_014765), and the MYR sequence: ATGGGGAGTAGCAAGAGCAAGCCTAAGGACCCCAGCCAGCGC, cloned into pENTR1A w48-1 (Eric Campeau). Gateway recombination using pCS2+6xMyc DEST and pLenti6.2 DEST cLAP provided by M. Nachury (Stanford University, Stanford, CA), and pcDNA C-term 6xMyc DEST (pTS2608) were used to produce Myc-FOP-FGFR1 (pTS2305), Myc-FOP-FGFR1*^K259A^* (pTS2505), Myc-FOP-FGFR1*^V74F/E97K^* (pTS2419), FOP-FGFR1-LAP (pTS2306), FOP-FGFR1*^K259A^*-LAP (pTS2807), FOP-FGFR1*^V74F/E97K^*-LAP (pTS2808), Myc-cFGFR1 (pTS2319), Myc-idFGFR1 (pTS2345), Myc-PACT-idFGFR1 (pTS2347), MTS-idFGFR1-Myc (pTS2682), and MYR-idFGFR1-Myc (pTS2642).

### Antibodies

Mouse (M) α–polyglutamylated tubulin (GT335; C. Janke, Centre de Recherches de Biochemie Macromoléculaire) was used at 1∶5000 and M α–γ-tubulin (GTU-88; Sigma-Aldrich) at 1∶1000. Rabbit (Rb) α-GFP antibody was previously described [40]. M α-Myc (9E10; Sigma-Aldrich): 1∶500 for IF, 1∶2000 for WBx. Mouse α-PY (4G10; Millipore): 1∶1000. Rb α-p38 (C-20; Santa Cruz Biotechnology, Inc.): 1∶5000. Rb α-phospho-PLCγ1 (cat. #2821; Cell Signaling Technology, Inc.) and rb α-PLCγ1 (cat. #2822; Cell Signaling Technology, Inc.): 1∶1000. Rb α-Smo (cat. #38686, Abcam): 1∶1,000. Rb α-Arl13b (cat. # 17711-1-AP; ProteinTech): 1∶500. Rb α-IFT88 (gift from Bradley Yoder, University of Alabama at Birmingham, AL): 1∶1,000. CD45-FITC (cat. # 347463; BD Biosciences): 1∶100. Double labeling using primary antibodies from the same host species was previously described [41].

### Lentivirus production

Lentiviruses expressing GFP-tagged WT, KD, and localization mutant FOP-FGFR1 were made using the lentiviral transfer vectors described above. Recombinant lentivirus was produced by cotransfection of HEK293T cells with the transfer vector, packaging vector (pCMVDR8.74) and envelope vector (pMD2.VSVG) using the calcium phosphate coprecipitation method [42].

### Cell culture, transfection, and cell lines

hTERT-RPE-1 cells (ATCC CRL-4000) were cultured in DMEM/F12 50/50 medium (Cellgro) + 10% fetal bovine serum (Atlanta Biologicals). RPE-1 cells were transfected with Lipofectamine LTX (Invitrogen) for 24 h followed by 24 h incubation in DMEM/F12 50/50 medium +0.5% fetal bovine +10 nM AP20187 (ARGENT Regulated Homodimerization Kit Version 2.0; ARIAD Pharmaceuticals, Inc.), or the equivalent volume of 100% ethanol (vehicle). For stable cell lines, RPE-1 cells were infected with lentiviral supernatant for 24 h and expanded. FACS, described below, was used to isolate the GFP-positive cells.

### Western blotting, Immunofluorescence, and FACS

Cells were lysed in triton buffer (1% triton, 150 mM NaCl, 50 mM Tris pH 8) supplemented with Protease Inhibitor Cocktail Tablets (cat. #11836170001; Roche) and PhosSTOP Phosphatase Inhibitor Cocktail Tablets (cat. #04906845001; Roche). Insoluble material was pelleted for 5 min at 3.3×kg (6,000 rpm) followed by Bradford analysis and 25 μg protein loaded. For kinase efficiency quantifications, blots were visualized using fluorescence conjugated secondary antibodies and the Typhoon 9210 imaging system (GE Life Sciences). Analysis was performed using ImageQuant TL v2003.01 (GE Life Sciences) with Local Average background correction and background subtraction of non-specific bands. For WB images, blots were visualized using HRP conjugated secondary antibodies and exposure to film. For IF experiments, cells were grown on poly-L-lysine coated coverslips and fixed with −20°C methanol. Coverslips were blocked in PBS-BT. Coverslips were incubated in primary antibodies diluted in PBS-BT then fluorescence-conjugated secondary antibodies (Invitrogen) diluted 1∶1000 in PBS-BT. Coverslips were imaged using OpenLab 4.0.4 on an Axiovert 200M microscope (Carl Zeiss MicroImaging, Inc.) with a Plan-NEOFLUAR 100× (1.3 NA) objective. Images were captured using an Orca-ER cooled CCD camera (Hamamatsu) and processed using Photoshop (Adobe Systems). Flow cytometry and FACS were performed at the Stanford Shared FACS Facility using the Scanford and Vantoo instruments, respectively, and analyzed using FlowJo (Tree Star, Inc.).

### Statistical Analysis

All statistical analyses were conducted with unpaired, two-tailed, Student's t tests using three independent trials. Values with p<0.05 were considered statistically significant.

## Supporting Information

Figure S1 **Localization of WT FOP-FGFR1 and mutants**. RPE-1 cells transfected with WT Myc-FOP-FGFR1, kinase-dead Myc-FOP-FGFR1*^K259A^*, or centrosome localization mutant Myc-FOP-FGFR1*^V74F/E97K^*, fixed, and stained with antibodies against Myc (green) and glutamylated-tubulin (red). Scale bars: 10 μm; insets: 10× magnification.
(TIF)

Figure S2 **FOP-FGFR1** ***^V74F/E97K^*** **dimerizes** ***in vivo*** **, but not** ***in vitro*** **. (A)** Table showing combinations of constructs used in co-expression, co-immunoprecipitation (co-IP) experiments. **(B)** *In vitro* translation of constructs in reticulocyte lysate followed by assessment of Myc-tagged FOP-FGFR1 constructs in immunoprecipitates (IP) of GFP-tagged FOP-FGFR1 constructs. **(C)** Expression of constructs in RPE-1 cells followed by assessment of Myc-tagged FOP-FGFR1 constructs in IP of GFP-tagged FOP-FGFR1 constructs.
(TIF)

Figure S3 **PACT dimerization.** Myc- and GFP-tagged PACT fused to truncated FGFR1 (PACT-cFGFR1) or PACT fused to idFGFR1 with the addition of dimerization ligand AP20187 (PACT-idFGFR1) were *in vitro* translated followed by assessment of Myc-tagged PACT constructs in immunoprecipitates (IP) of GFP-tagged PACT constructs.
(TIF)

Figure S4 **Ciliogenesis in cells expressing targeted idFGFR1. (A)** RPE-1 cells transfected with Myc-FOP-FGFR1, Myc-PACT-idFGFR1, or MTS-idFGFR1-Myc, incubated in low serum medium with dimerization ligand for 48 h, fixed, and stained with antibodies against Myc (green) and glutamylated tubulin (red). DNA is stained using DAPI (blue). Scale bars: 10 μm; insets: 10× magnification.
(TIF)

Figure S5 **Arl13b and IFT88 localization in CML cells containing protrusions. (A)** Primary human CML cells or normal PBMCs stained with antibodies against Arl13b (green) and glutamylated tubulin (red). **(B)** Primary human CML cells or normal PBMCs stained with antibodies against IFT88 (green) and glutamylated tubulin (red). **(C)** Primary human CML cells stained with antibodies against Smo (green) and Arl13b (red). DNA is stained using DAPI (blue), scale bars: 5 μm, white arrows: cell protrusions.
(TIF)

Figure S6 **Models for effect of centrosome protein fusion partner on kinase signaling.** **(A)** Targeting of kinases to the centrosome results in decreased mobility of the kinase, which can more effectively interact with diffusing substrate resulting in greater phosphorylation of normal kinase substrates and increased downstream signaling. **(B)** If kinases substrates are themselves concentrated at the centrosome, localization of the kinase results in increased substrate availability, resulting in increased phosphorylation and increased downstream signaling.
(TIF)

Table S1 **Clinical characteristics of CML and AML patient samples.**
(DOCX)
