# Supplementary figures and images for: Centrosome-Kinase Fusions Promote Oncogenic Signaling and Disrupt Centrosome Function in Myeloproliferative Neoplasms
Source: PLoS One. 2014 Mar 21;9(3):e92641. doi: 10.1371/journal.pone.0092641 (PMC3962438; doi:10.1371/journal.pone.0092641)

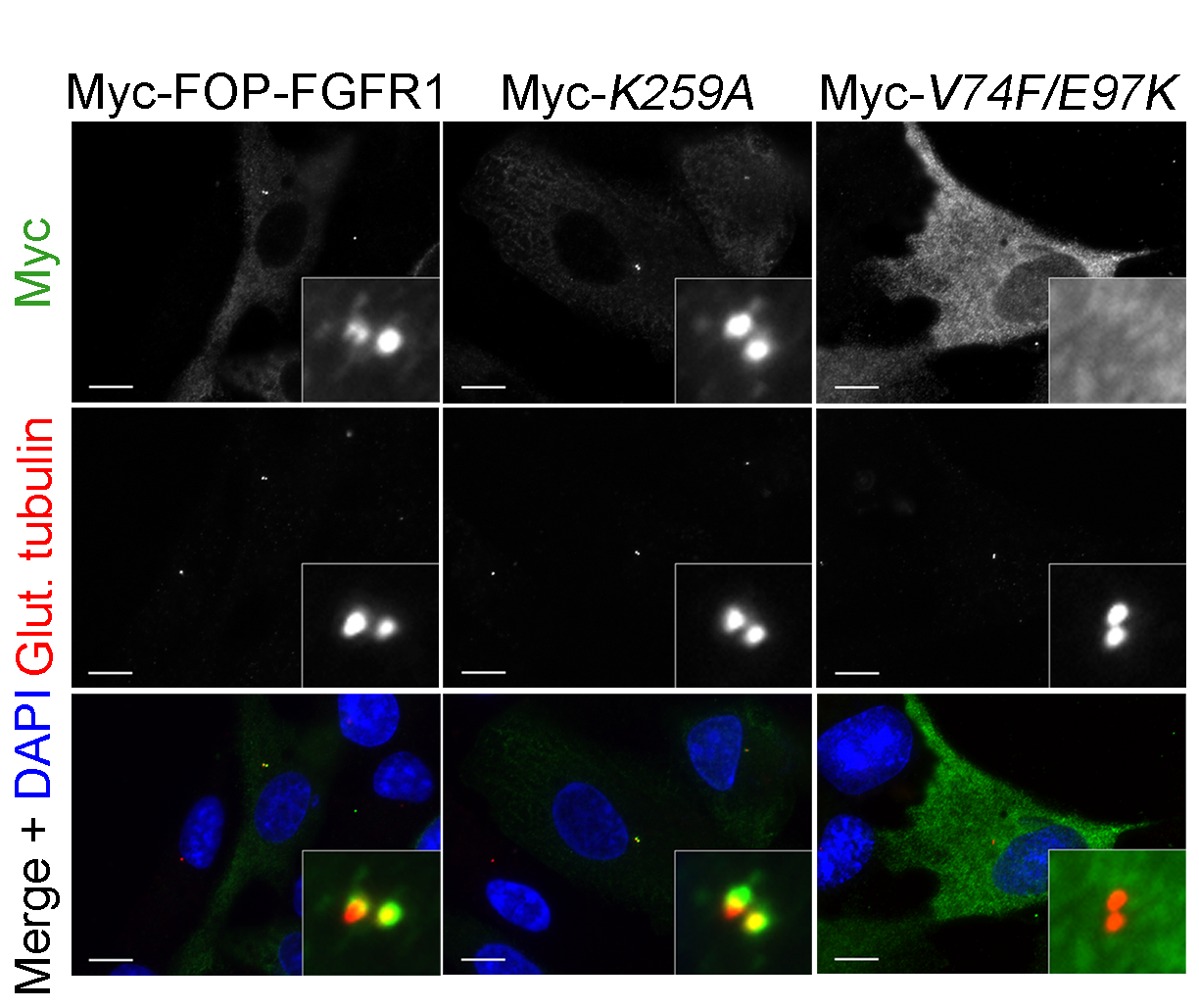

Supplement: Figure S1 — Localization of WT FOP-FGFR1 and mutants. RPE-1 cells transfected with WT Myc-FOP-FGFR1, kinase-dead Myc-FOP-FGFR1K259A, or centrosome localization mutant Myc-FOP-FGFR1V74F/E97K, fixed, and stained with antibodies against Myc (green) and glutamylated-tubulin (red). Scale bars: 10 μm; insets: 10× magnification. (TIF) [file pone.0092641.s001.tif]

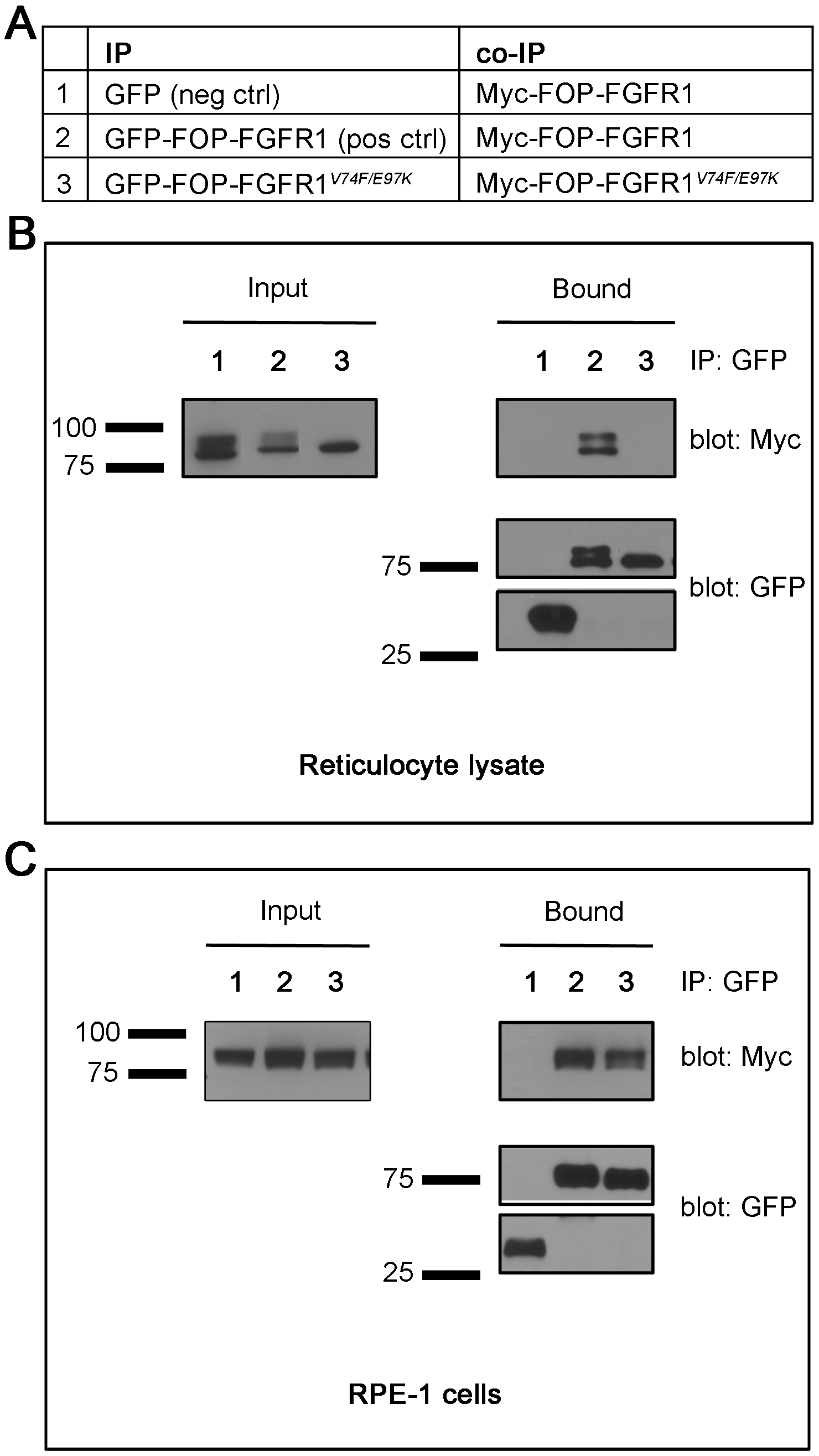

Supplement: Figure S2 — FOP-FGFR1 V74F/E97K dimerizes in vivo , but not in vitro . (A) Table showing combinations of constructs used in co-expression, co-immunoprecipitation (co-IP) experiments. (B) In vitro translation of constructs in reticulocyte lysate followed by assessment of Myc-tagged FOP-FGFR1 constructs in immunoprecipitates (IP) of GFP-tagged FOP-FGFR1 constructs. (C) Expression of constructs in RPE-1 cells followed by assessment of Myc-tagged FOP-FGFR1 constructs in IP of GFP-tagged FOP-FGFR1 constructs. (TIF) [file pone.0092641.s002.tif]

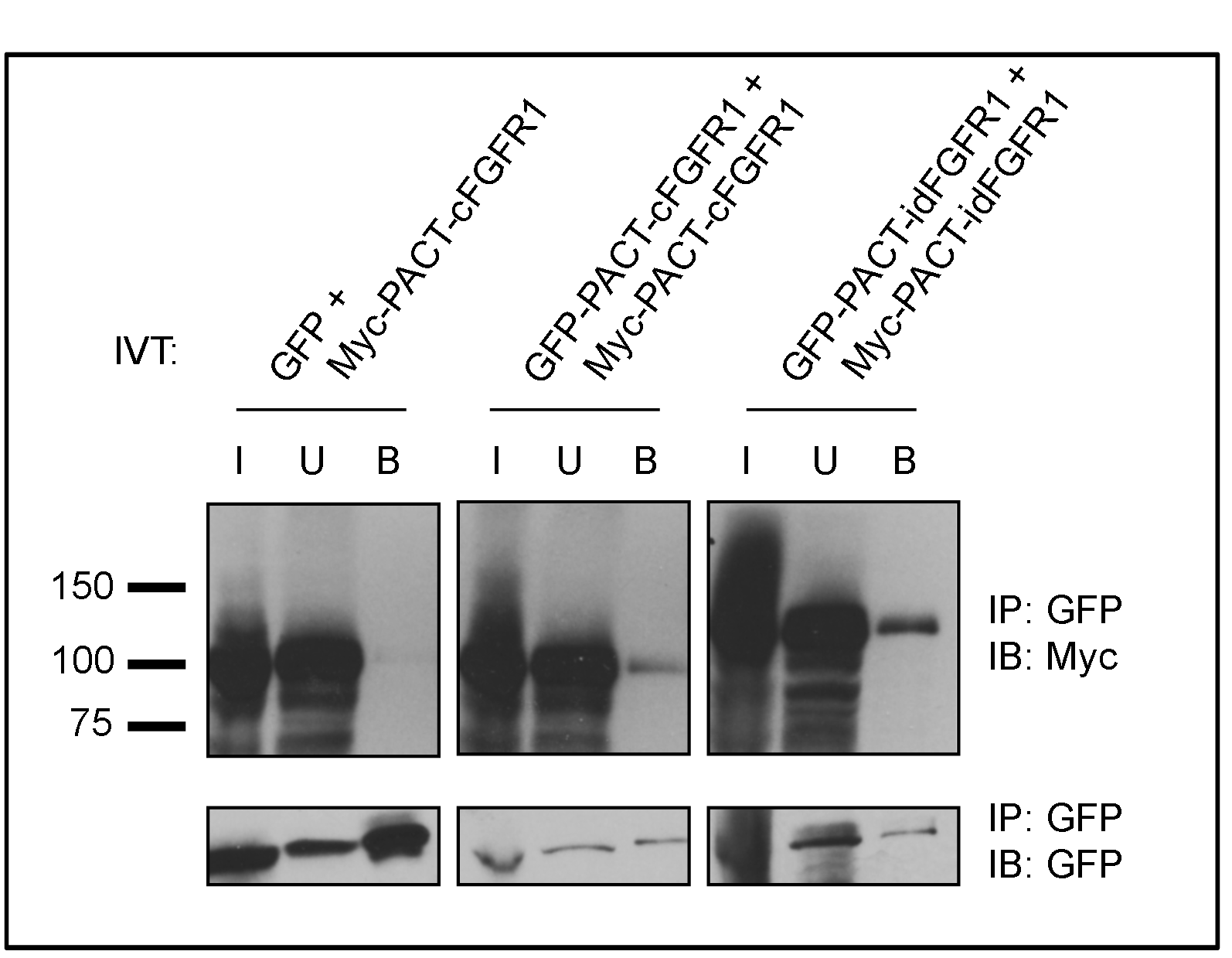

Supplement: Figure S3 — PACT dimerization. Myc- and GFP-tagged PACT fused to truncated FGFR1 (PACT-cFGFR1) or PACT fused to idFGFR1 with the addition of dimerization ligand AP20187 (PACT-idFGFR1) were in vitro translated followed by assessment of Myc-tagged PACT constructs in immunoprecipitates (IP) of GFP-tagged PACT constructs. (TIF) [file pone.0092641.s003.tif]

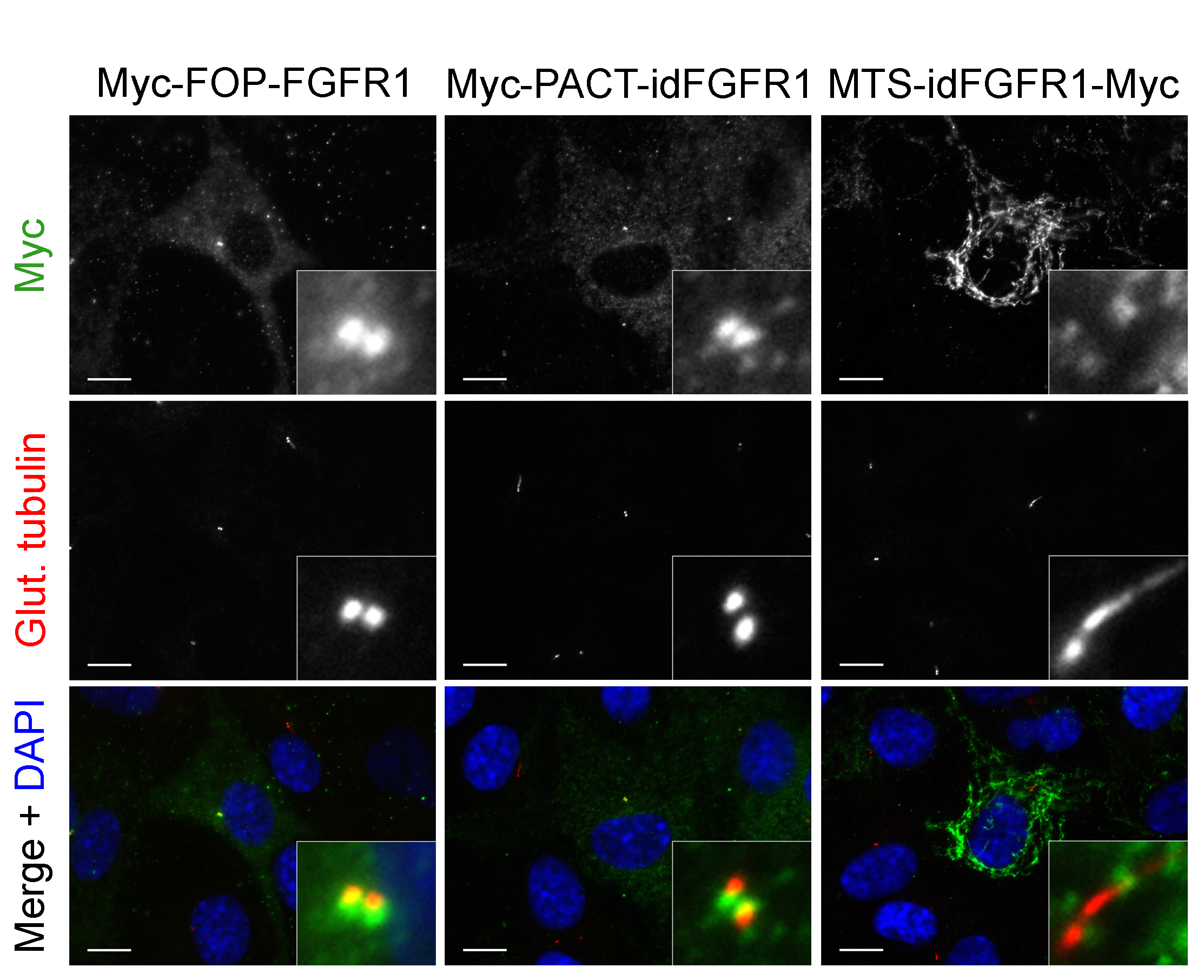

Supplement: Figure S4 — Ciliogenesis in cells expressing targeted idFGFR1. (A) RPE-1 cells transfected with Myc-FOP-FGFR1, Myc-PACT-idFGFR1, or MTS-idFGFR1-Myc, incubated in low serum medium with dimerization ligand for 48 h, fixed, and stained with antibodies against Myc (green) and glutamylated tubulin (red). DNA is stained using DAPI (blue). Scale bars: 10 μm; insets: 10× magnification. (TIF) [file pone.0092641.s004.tif]

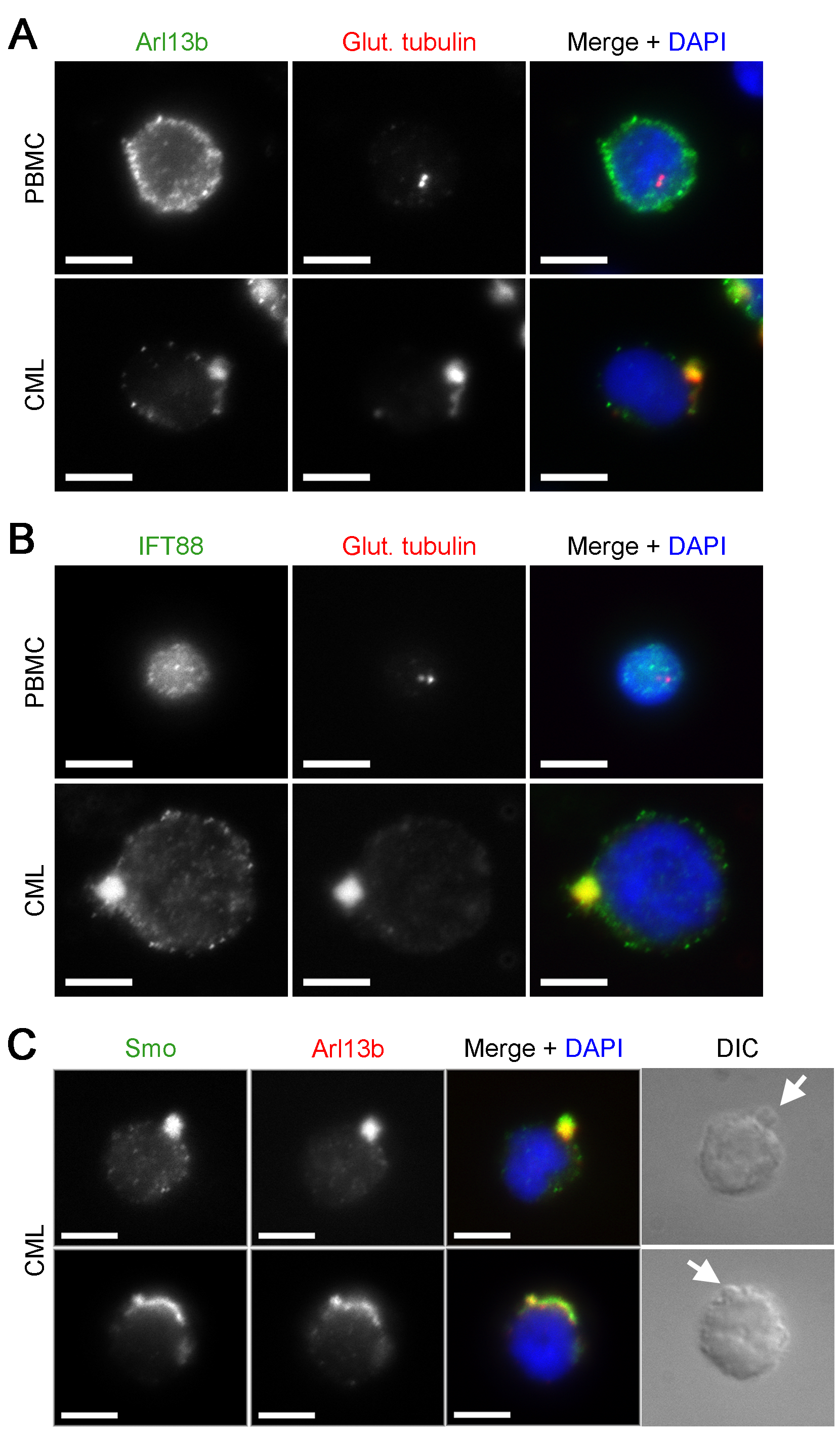

Supplement: Figure S5 — Arl13b and IFT88 localization in CML cells containing protrusions. (A) Primary human CML cells or normal PBMCs stained with antibodies against Arl13b (green) and glutamylated tubulin (red). (B) Primary human CML cells or normal PBMCs stained with antibodies against IFT88 (green) and glutamylated tubulin (red). (C) Primary human CML cells stained with antibodies against Smo (green) and Arl13b (red). DNA is stained using DAPI (blue), scale bars: 5 μm, white arrows: cell protrusions. (TIF) [file pone.0092641.s005.tif]

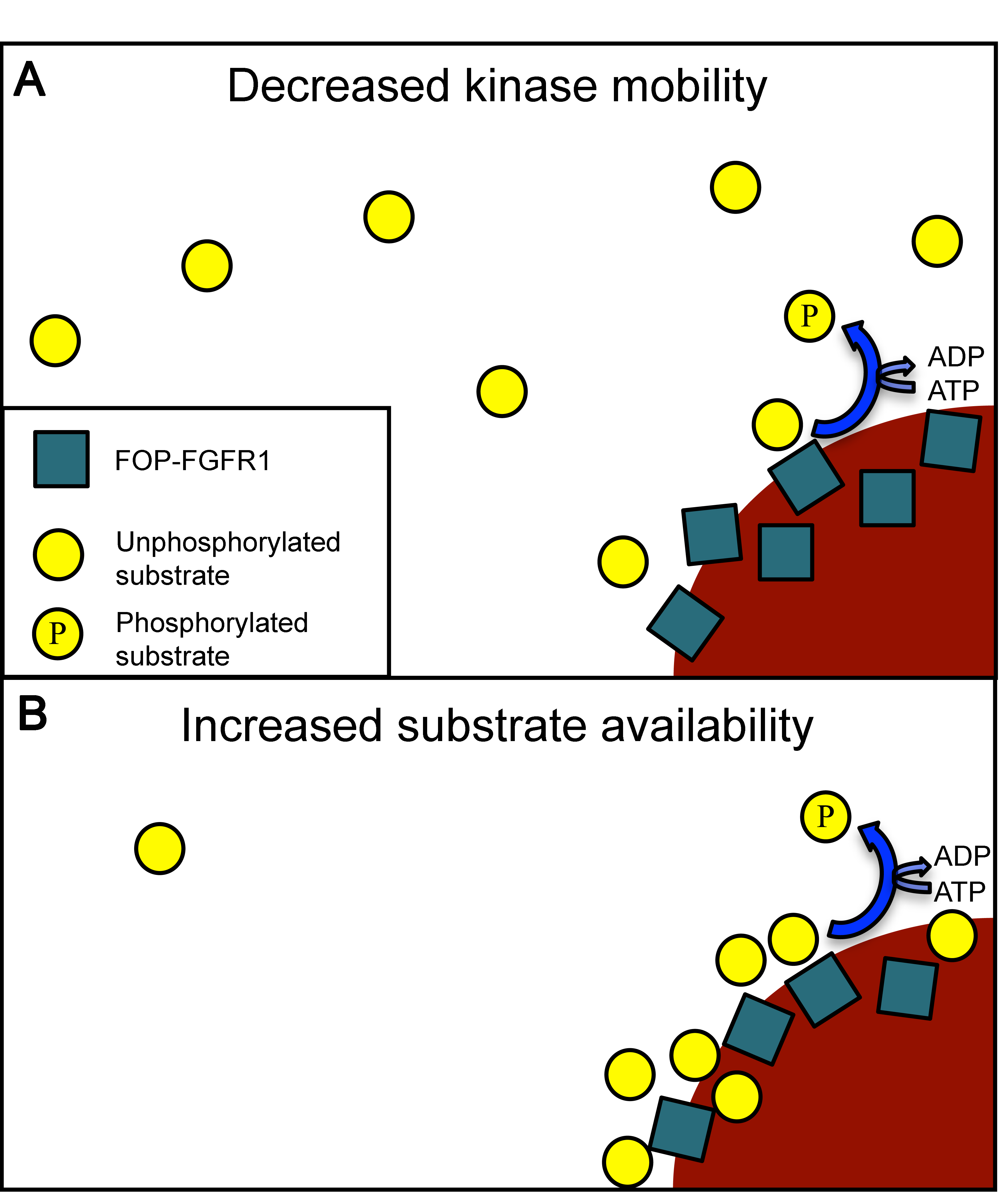

Supplement: Figure S6 — Models for effect of centrosome protein fusion partner on kinase signaling. (A) Targeting of kinases to the centrosome results in decreased mobility of the kinase, which can more effectively interact with diffusing substrate resulting in greater phosphorylation of normal kinase substrates and increased downstream signaling. (B) If kinases substrates are themselves concentrated at the centrosome, localization of the kinase results in increased substrate availability, resulting in increased phosphorylation and increased downstream signaling. (TIF) [file pone.0092641.s006.tif]
