# Supplementary material for: Centrosome-Kinase Fusions Promote Oncogenic Signaling and Disrupt Centrosome Function in Myeloproliferative Neoplasms
Source: PLoS One. 2014 Mar 21;9(3):e92641. doi: 10.1371/journal.pone.0092641 (PMC3962438; doi:10.1371/journal.pone.0092641)
Supplement: Table S1 — Clinical characteristics of CML and AML patient samples. (DOCX) [file pone.0092641.s007.docx]

**Table S1. Clinical characteristics of CML and AML patient samples.**

| **Sample ID** | **Disease classification** | **Age** | **Sex** | **Disease Status** | **Cytogenetics** |
| --- | --- | --- | --- | --- | --- |
| SU034 | CML | 49 | Male | Chronic-Phase | t(9;22) |
| SU038 | CML | 43 | Male | Chronic-Phase | t(9;22) |
| SU287 | CML | 23 | Male | Chronic-Phase | t(9;22) |
| SU266 | AML | 64 | Male | De novo | inv(3)(q21q26) |
| SU320 | AML | 68 | Male | De novo | No analyzable metaphases |
